# Effectiveness of electropolishing step on elimination of upstream manufacturing material residues on metallic orthopaedic medical device

**DOI:** 10.3389/fmedt.2026.1803156

**Published:** 2026-05-28

**Authors:** Karolina Godlewska, Christopher Joseph Graham, Michael Frohbergh, Srivaths Kalyan, Rasagnya Viswanadha, Philippe Hasgall

**Affiliations:** 1 Zimmer Switzerland Manufacturing GmbH, Winterthur, Switzerland; 2 Zimmer, Inc., Warsaw, IN, United States; 3 Exponent Inc., Menlo Park, CA, United States

**Keywords:** biocompatibility, biological assessment, electropolishing, manufacturing materials residues, reset

## Abstract

Orthopaedic medical device manufacturing often utilizes manufacturing materials or processing aids that can remain on device surfaces as residues and result in leachable chemicals to which patients can be exposed upon contact with the device. Manufacturing steps that modify a device surface through electrochemical means such as electropolishing can remove manufacturing material residues and be considered as surface “reset” in the manufacturing process. In this report, a retrospective analysis of chemical characterization data gathered for medical devices subjected to electropolishing steps during the manufacturing process is summarized. Review of the extractables data from metallic medical devices demonstrated that electropolishing operations effectively remove the residues originating from manufacturing materials utilized upstream of electropolishing. Based on this retrospective review, electropolishing can serve as a surface “reset” point in the manufacturing process in biological safety evaluations, thereby reducing the overall testing requirements, including animal testing, in alignment with the ISO 10993 framework.

## 1 Introduction

Medical devices aim to assist patients with underlying medical conditions or pathologies as well as provide clinicians and healthcare providers with tools and instruments to improve the methods by which patients are treated or cared for. Examples of medical devices include orthopedic implants, trauma implants and associated instrumentation. Implants as well as instruments must meet certain criteria to support biocompatibility, mechanical integrity, and other functions depending on the clinical use. These devices typically undergo a variety of surface treatments to enhance different desired features, such as corrosion resistance, surface energy, wettability, hardness, and biocompatibility.

The ISO 10993 series comprise international standards that guide the evaluation of the biological safety of medical devices. Part 1 (1) of the ISO 10993 standard series provides an overview of biocompatibility requirements, device categorization, biological effects to be considered, and the risk-based approach for assessment. This risk-based approach requires manufacturers to systematically review all relevant data on the raw materials, process flows, historical pre-clinical data, and clinical data. Gaps are identified to determine whether the available data are sufficient to complete a biological risk assessment or whether additional data need to be generated to close the identified gaps.

**Figure 1:**
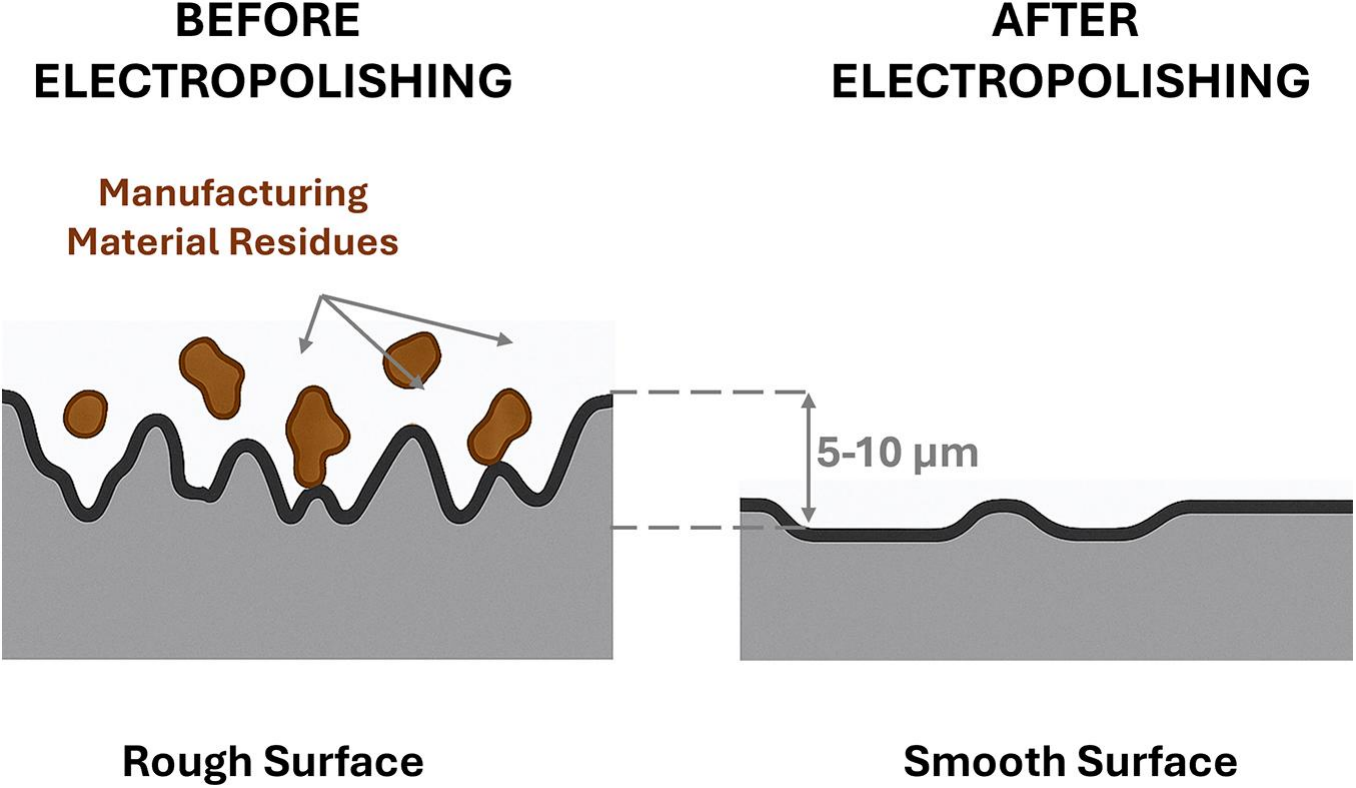
Depiction of contamination removal during electropolishing.

In orthopedic devices, base materials of construction for implants and instruments are typically polymeric, metallic or ceramic. Stainless steel (e.g., bone screws; washers; scalpels), titanium (e.g., dental implants; bone screws; spinal plates) and aluminum (e.g., aluminum ophthalmic eye shield; perforated instrument cases used to store and sterilize instruments, orthopedic depth gauge) are commonly used materials of construction for implant and instrument devices, and various processes (e.g., electropolishing, anodization, passivation) are used for surface modification. One widely used surface modification process is electropolishing. Electropolishing is an electrochemical process that removes material from the device surface, with a greater amount being removed from peaks on the surface profile, thereby reducing surface roughness (2). In a typical electropolishing setup, the component to be electropolished (i.e., medical device) acts as the anode, while the counter electrode (i.e., the metal on which material is intended to be plated, or electroplated) acts as the cathode. Removing microscopic burrs, embedded contaminants, and free iron from the surface during the electropolishing process results in a smoother, cleaner, and more corrosion-resistant finish, as shown in Figure 1. Electropolishing typically removes between 5 and 10 µm of the surface (3), and by smoothing the surface, removing surface residues, and improving corrosion resistance, it can improve the biocompatibility of the metallic medical devices.

The impact of electropolishing on the biocompatibility of medical devices (implants and instruments alike) has been demonstrated previously (4–7). Cattaneo et al. (4) investigated the effectiveness of braided, electropolished Nitinol (a titanium alloy) microwires on biocompatibility. The study assessed biochemical processes, such as hemostasis, inflammation, and cell proliferation by evaluating protein adsorption, thrombogenicity, human umbilical vein endothelial cell (HUVEC) behavior, and ICAM-1 expression. In all cases, the electropolished surfaces showed improved trends (e.g., lower thrombogenicity; reduced protein absorption; improved endothelialization; changed surface chemistry with a more stable layer of titanium oxide and titanium oxynitride; lower or comparable ICAM-1 expression) over the control, non-electropolished sample, indicating a higher level of biocompatibility of the electropolished device. Duan et al. (5) investigated the use of a specialized electropolishing method, in which the sample is electropolished with a higher voltage. While this is a slight variation on traditional electropolishing, the mechanism of polishing is identical in terms of modifying the surface. This study compared osteoblast growth on a non-electropolished 316LVM stainless steel surface (control) with an electropolished 316LVM surface. The results showed that the growth and spreading of osteoblasts on the electropolished surface was more prolific than the spreading of osteoblasts on the control surface, indicating that electropolished surfaces were more conducive to cell growth, spreading, and migration. Wang et al. (2) investigated the use of environmentally friendly electrolytes to perform electropolishing on Nitinol tubes. While this study did not explicitly perform *in vitro* or *in vivo* biocompatibility testing, the study included water contact angle measurements for both untreated and electropolished Nitinol tubes. The contact angle increased by 32.8°, indicating higher hydrophilicity, and thus, better potential for cell adhesion and biocompatibility. Omanovic et al. (7) investigated the different cell voltages during electropolishing on 316L stainless steel to identify the optimal operational parameters such as electrolyte composition, temperature, current density, applied potential, polishing time, agitation and cathode material. Their experiments with platelet-rich plasma demonstrated that electropolishing surfaces exhibit a much lower degree of thrombogenicity. In all cases, the electropolished surfaces demonstrated an improved level of biocompatibility of the electropolished surfaces compared to non-electropolished controls.

Surface finishing is known to influence not only the physicochemical quality of metallic implants but also their biological performance, as has been particularly demonstrated for dental implants (8). In general, the literature indicates that biological response is governed by a combination of roughness, topography, wettability, and surface chemistry rather than by roughness alone (9). Highly smooth finishes produced by electropolishing are beneficial for reducing surface defects, inclusions, and corrosion susceptibility, and may be advantageous where a cleaner and more chemically homogeneous surface is desired. However, comparative studies and recent reviews also indicate that moderately rough or micro/nanostructured surfaces generated by approaches such as blasting, acid etching, anodization, or controlled porous texturing can promote stronger early osteoblast response, favorable macrophage polarization, and faster osseointegration than mirror-like surfaces, particularly when roughness is combined with hydrophilic surface chemistry (10, 11). Accordingly, electropolishing should be viewed as one valuable finishing strategy within a broader design space, whereas the optimal surface condition depends on the intended clinical function and the desired balance between surface integrity, corrosion resistance, and bone-implant integration.

While there is limited literature discussing the impact of electropolishing on the surface finish of medical devices, this is just one aspect of biocompatibility. Another, less extensive study aspect is the removal -or persistence- of manufacturing process residues from the surface of these devices. To the best of the authors’ knowledge of the publicly available literature, no studies have assessed the use of electropolishing as a manufacturing step that ‘resets’ the device surface thereby mitigating the biocompatibility risk arising from upstream manufacturing material residues.

Throughout the manufacturing process of medical devices, various manufacturing materials are used. Analytical methods can be used to detect the presence of residues from manufacturing materials used during production of the final medical device. ISO 10993-18 provides an array of chemical characterization techniques to identify a wide range of chemicals that may potentially leach during clinical use (12). As stated, in ISO 10993-18, an extractable is defined as chemical substances that can be released from a medical device or its materials when exposed to laboratory extraction conditions and extraction vehicles. A retrospective analysis of chemical characterization evaluations performed according to ISO 10993-18 to support the biological safety of their various products has been completed to assess the impact of surface modification steps on the extractables profile of the final, finished devices.

The hypothesis of this study is that the electropolishing step within the process flow may effectively ‘reset’ the device surface by removing contaminants and surface irregularities as illustrated in Figure 1, thereby mitigating the risk of upstream manufacturing material residues. The retrospective data analysis of chemical characterization datasets facilitates addressing the hypothesis by establishing an association between extractables found in chemical characterization testing and manufacturing materials used to manufacture test articles.

## 2 Material and methods

### 2.1 Selection of datasets for retrospective analysis

One hundred twelve chemical characterization data sets on all-metallic orthopaedic devices generated between 2021 and 2025 were available for retrospective data analysis. Datasets involving anodized or passivated surface modifications were excluded to avoid confounding effects associated to differing surface treatments, and to maintain alignment with the scope of this study, which is focused exclusively on electropolished devices. The evaluation of anodized and passivated devices nonetheless remains of potential interest and may warrant dedicated investigation in future work.

Manufacturing process flows for each test article were classified based on their initial surface modification operation into one of the following five groups: coated, blasted, polished, electropolished and non-modified. The steps in each process flow for metallic orthopaedic device were analyzed, and the first surface modification operation determined the group assignment. For example, a process flow that included mechanical polishing as the first modification was categorized under ‘Polished’ group. Each dataset was only assessed as part of one of the classifications shown in Table 1.

**Table 1:** Surface modification categorization.

Name of surface modification group*	Description of surface modification group	Number of tested devices in surface modification group	Number of evaluated chemical characterization datasets
Coated	Metal coated devices with a porous surface, such as porous plasma sprayed.	13	0
Blasted	Metal devices with a ‘Blasting’ operation present in the process flow.	41	0
Polished	Metal devices with a mechanical ‘Polished’ operation present in the process flow.	14	2
Electropolished	Metal devices with ‘Electropolishing’ operation present as the first surface modification in the process flow.	9	9
Non-modified	Metal devices with no surface modification included in the process flow.	4	4

* The first surface modification present in their process flow was used during classification and sub-grouping.

### 2.2 Microsoft power BI analysis of selected chemical characterization datasets

To compare extractable data from devices with the various surface modifications, exposure (i.e., amount of each extractable detected) was normalized to the surface area of the device. The following calculations were performed to compare three relevant exposure parameters:

(1) Substance level exposure

For each reported extractable, the exposure in µg/cm^2^ was calculated using the following formula:

$$Substance\;exposure\;\left[\frac{\mu g}{cm^{2}}\right]=\frac{Substance\;exposure\;[\mu g/device]}{Surface\;area\;[cm^{2}/device]}$$

(2) Total Device Exposure

Total device exposure for all extractables in units of µg/cm^2^ was determined for each tested device using the following formula:

$$\;Total\;device\;exposure[\mu g/cm^{2}]\;=\overset{n}{\underset{i=1}{\sum }}\;Substance\;Exposure_{i}[\mu g/cm^{2}]$$

(3) Surface Modification Group Statistics

Devices were grouped by initial surface modification type. For each group, the maximum, minimum, median, population standard deviation and standard deviation of sample values of total device exposure were calculated to characterize the distribution and facilitate comparison of extractable exposure across different surface modifications.

### 2.3 Retrospective analysis

The aim of this retrospective analysis is to identify correlations between extractables found in chemical characterization tests of electropolished, polished, blasted, coated and non-modified devices, and directly contacting manufacturing materials used throughout their manufacturing processes. The objective is to determine whether extractables originate from manufacturing materials used in steps prior to or after the analyzed surface modification. The five surface modification groups (electropolished, polished, blasted, coated and non-modified devices) were initially analyzed; and based on analysis, three groups (electropolished, polished, and non-modified devices) were selected for subsequent analysis. The analysis described in Section 2.2 included the full group of devices with either full and partial surface modifications. In contrast, the subsequent analysis in Section 2.3 was limited to devices that were fully modified across the entire device surface. This approach was taken to reduce variability associated with partial surface treatments and inclusion of multiple material types.

The analytical evaluation threshold (AET) defines the experimental based limit above which extractables should be reported in a chemical characterization screening study. The AET (12) is based on the dose-based threshold (DBT), which is the dose limit for organic compounds, with the exception of certain high potency toxicity compounds termed cohort of concern (CoC), below which the risk of toxicity of a leachable is deemed negligible. To derive the AET, the DBT is modified by device and analytical parameters, including clinical exposure, volume of device extract, and analytical method uncertainty. Only compounds detected above study specific AETs were evaluated. Correlations between extractables detected in chemical characterization reports and manufacturing process flows of tested articles were determined by considering available information about the manufacturing materials, such as manufacturer declarations (e.g., safety data sheets), known reaction and breakdown products, and common industrial uses.

**Figure 2:**
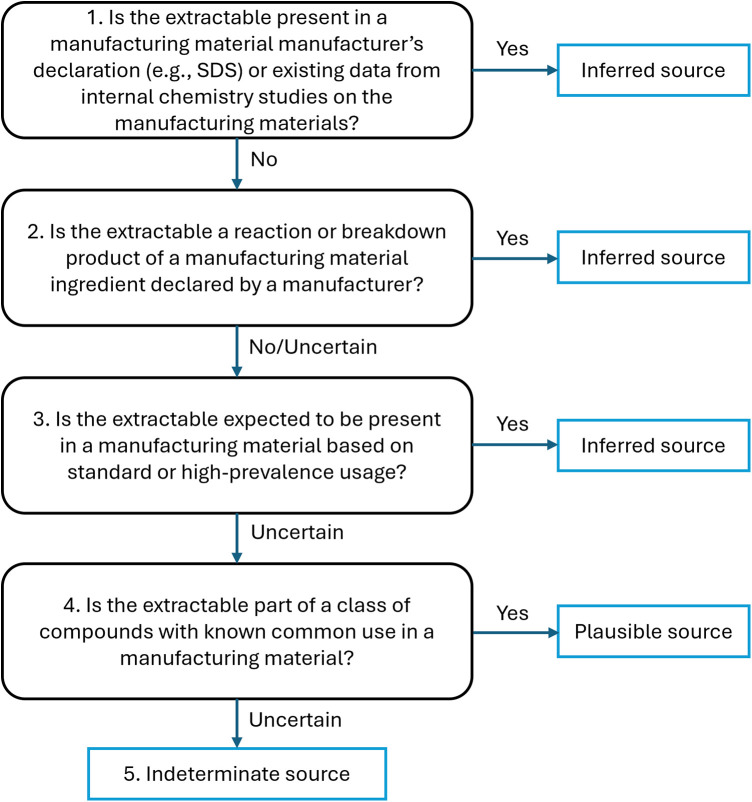
Extractables to Manufacturing Material Correlation Decision Tree.

For correlation of extractables with a manufacturing material, a priority-based decision approach was followed, which is represented in Figure 2. Briefly, manufacturer declarations (e.g., safety data sheets, product data sheets, and regulatory data sheets) together with internal data on manufacturing materials (i.e., chemical analysis of manufacturing materials) were given precedence for the evaluation. If an extractable was identified and explicitly listed by the manufacturer for the manufacturing material, or if existing chemical data confirmed the presence of the extractable in the manufacturing material, that manufacturing material was assumed to be the source. In some instances, manufacturer declarations (e.g., safety data sheets) included mixtures (e.g., CASRN 61791-14-8 Amines, coco alkyl, ethoxylated, which is a mixture of ethoxylated coconut oil fatty amines), so individual extractables within these mixtures were also given precedence. Since the composition of the manufacturing materials is not always available, further evaluations were necessitated. An assessment of whether the extractable can be attributed to an expected breakdown or reaction products of a known ingredient in the manufacturing material was performed. This would include, for example, ethoxylated fragments that could be attributed to known ethoxylated detergents. In those cases, the manufacturing material was inferred to be the source based on available evidence and expert judgement. If attribution was not possible, the extractable was evaluated to determine whether it is an expected ingredient that has not been explicitly declared. For example, erucamide (CASRN 112-84-5) is a very common slip agent used in device packaging substrates (e.g., LDPE). Polymer resin manufacturers may declare the presence of a slip agent but not specify the compound. Erucamide is a very common slip agent, and in the case of the known use of LDPE as a device package and the identification of erucamide as an extractable, the association would be very likely. If no direct correlations could be established, sources with common industrial applications for the relevant classes of compounds were identified. For example, antioxidants such as Irgafos® 168 (CASRN 31570-04-4) are commonly used in polymer production, and long chain alkane/alkenes would be common components of base oils. To further support the correlations mentioned above (especially when no direct correlation was identified), chemical characterization data from other devices using the same manufacturing materials were referenced to identify common extractables. Finally, if no plausible source could be determined, the manufacturing material origin source of the extractable compound was considered indeterminate.

After tracing the origin of the extractable substance to the manufacturing material, the specific manufacturing step(s) in which the material was used was identified. This allowed the determination of whether extractables originating from manufacturing materials were used in manufacturing steps prior to the analyzed process step (electropolishing or polishing or cleaning) or originating from that analyzed step or subsequent operations. If an extractable was associated with both pre- and post- analyzed process steps, both associations were reported.

The polished and non-modified test articles served as control comparators for assessing the effect of electropolishing. The polished test articles were introduced as a control group to verify whether the proposed correlation method can associate extractables with manufacturing steps prior to electropolishing. Only devices with end-of-line processing equivalent to that used for the electropolished devices, characterized by using identical final cleaning detergent and identical biological upgrade (i.e., microbial bioburden reduction method) were included in the polished control group to eliminate variation related to detergent efficiency during final cleaning.

The non-modified test articles were introduced as a second control group to determine if the proposed correlation method between extractables, manufacturing materials and steps in process flow can associate any extractables with pre-cleaning steps. Since test articles in the non-modified group include only machining and cleaning steps the results of the associations will show if a cleaning step alone is sufficient to remove upstream manufacturing residues.

### 2.4 Selection criteria

Prior to conducting the retrospective analysis the following selection criteria were applied:

(I) Each evaluated electropolishing step was confirmed to meet external electropolishing specifications. Specifically, ASTM B912 (3) defines that during the electropolishing process, 5–10 µm of the surface is typically removed and up to 50 µm can be removed for additional smoothing.
(II) Each evaluated device was confirmed to be fully electropolished or polished, i.e., the entire surface area of device was modified. Devices that were only partially electropolished or polished were excluded from this analysis.
(III) For the polished devices group, devices that use the same final cleaning detergent and biological upgrade method as the electropolished group were selected.
(IV) Only organic substances in the chemical characterization datasets were analyzed since they would not be associated with the device raw materials and thus are indicative of manufacturing material residues.

After application of the above selection criteria, nine electropolished devices, two polished devices, and four non-modified devices met the criteria and were selected for analysis (Table 1). Electropolished devices were constructed from different grades of stainless steels, such as 1.4441 stainless steel, 316 Low Vacuum Melting (LVM) stainless steel, 316L stainless steel, BioDur® 108C stainless steel, or 22Cr-13Ni-5Mn stainless steel. Polished devices were constructed from Cobalt-Chromium-Molybdenum (CoCrMo) alloy or Titanium-6Aluminum-4Vanadium (Ti-6Al-4 V) alloy. Non-modified devices were made from Titanium-6Aluminum-7Nobium (Ti-6Al-7Nb) alloy or Ti-6Al-4 V alloy.

## 3 Results

**Figure 3:**
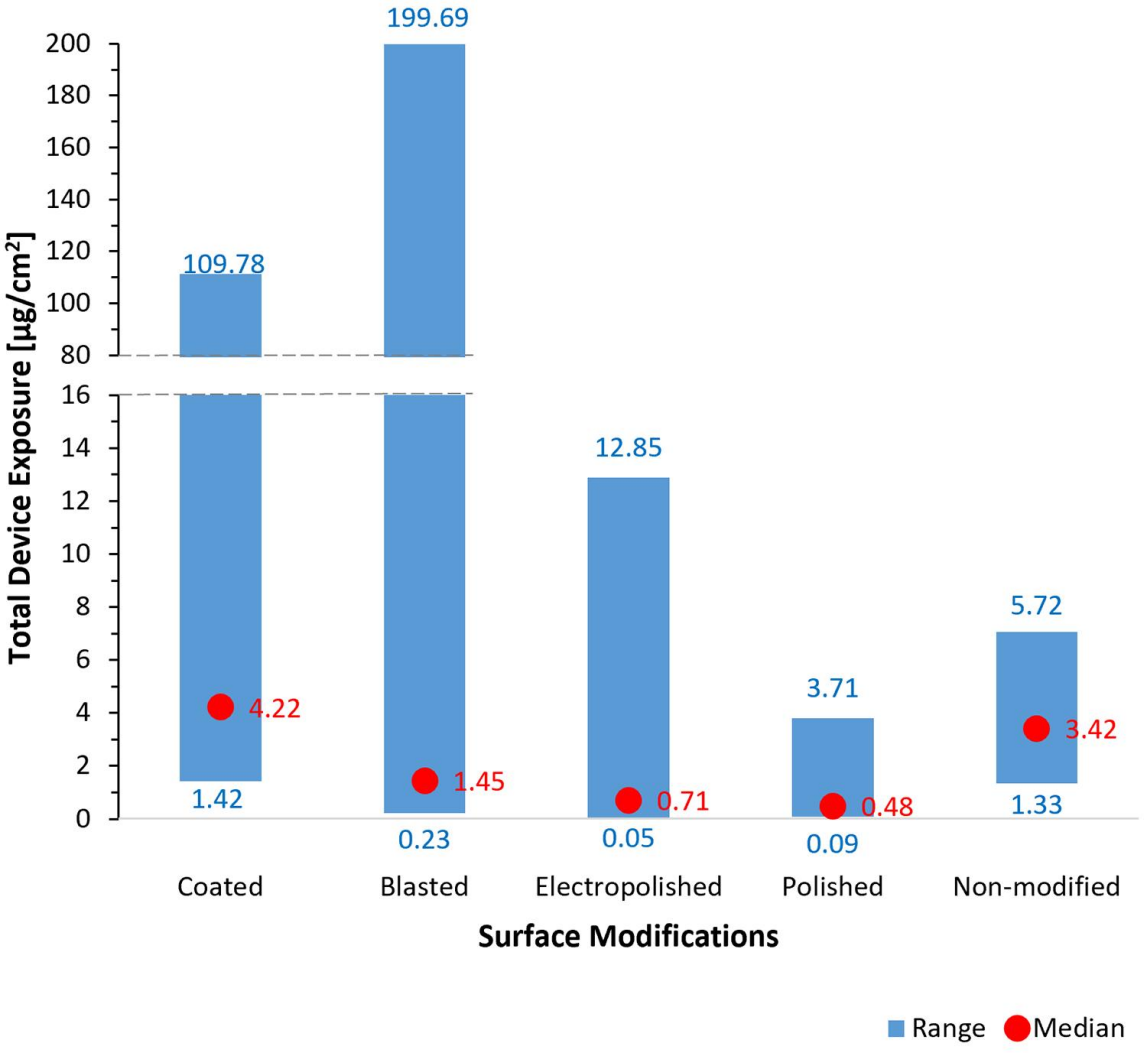
Median (red points) and the range (blue bars) of Total Device Exposure for each surface modification.

Five surface modification groups listed in Table 1 were plotted based on the total device exposure. The histogram in Figure 3 shows the calculated range (blue bars) and median value (red points) of total device exposure for each surface modification group.

Among analyzed surface modification groups, the ranges for total device exposure were lower (i.e., less than 10 fold) for electropolished, polished surface modifications and non-modified surfaces compared to coated and blasted surface modifications. The median total exposure values for electropolished and polished surface modifications were lower (below 1 µg/cm^2^) compared to coated, blasted surface modifications and non-modified groups for which median total exposure values varied between 1.45 and 4.22 µg/cm^2^. When considering both a low median exposure and a low overall exposure range, the data indicate that electropolished and polished surface modifications may be more effective at removing manufacturing residuals and thus warranted further evaluation in this analysis.

The results of the statistical analysis are presented in Table 2. As shown, the electropolished, polished, and non-modified device groups exhibit approximately one order of magnitude lower sample standard deviations compared to all other analyzed groups. Specifically, the sample standard deviations were 4.36, 1.06, and 2.06 µg/cm^2^ for the electropolished, polished, and non-modified devices, respectively. These lower standard deviations indicate that these three surface modifications are more consistent at the removal of manufacturing residues. Tables 3 through 5 summarize extractables detected on electropolished, polished, and non-modified devices, respectively. Table 4 shows examples of substance association to potential manufacturing materials and manufacturing steps in the process flows of analyzed devices. Full data of extractables found on electropolished, polished and non-modified devices is provided in Supplementary Table S1, S2, S3, respectively.

**Table 2:** Statistic analysis for analyzed surface modification groups.

Surface Modification	Median	Min Value	Max Value	Population Standard Deviation	Standard Deviation of Sample
Coated	4.22	1.42	109.78	36.18	37.55
Blasted	1.45	0.23	199.69	30.67	31.06
Electropolished	0.71	0.05	12.85	4.11	4.36
Polished	0.48	0.09	3.71	1.02	1.06
Non-modified	3.42	1.33	5.72	1.84	2.06

**Table 3:** Summary of extractables detected above the AET on devices from electropolished devices group. If an extractable was associated with both pre- and post- electropolishing step, both associations were shown in the table below.

Test Article	Total number of organic extractables above AET	Number of extractables associated with manufacturing materials used		Number of extractables not associated with any manufacturing materials
		at electropolishing operation or at the post-electropolishing operations	at the pre-electropolishing operations	
1st test article	1	1	0	0
2nd test article	5	3	0	2
3rd test article	3	1	0	2
4th test article	1	1	0	0
5th test article	3	3	0	0
6th test article	3	2	0	1
7th test article	3	1	0	2
8th test article	11	0	0	11
9th test article	7	0	0	7

**Table 4:** Examples of substance association for all analyzed device groups.

Devices group	Extractables	Potential Source of Extractables	Association to manufacturing step
Electropolished	Polyethylene glycol (PEG) containing compound	Final cleaning detergent—because the final cleaning detergent is a mixture of PEG and alkyl chains. One ingredient of the final cleaning detergent is alkyl-PEG-ether phosphoric acid esters, Na-salt (CAS: 111798-26-6).	Post-electropolishing
	Caprolactam	The polyamide (PA) packaging—because caprolactam is a pre-cursor of PA, and the PA bag was used as the final packaging material.	Post-electropolishing
Polished	Short chain ketones, aldehydes, and acids	Final cleaning detergent—because the final cleaning detergent contains multiple short chain oxygenated aliphatic compounds, including ketones, esters, and carboxylic acid. Although the extractables linked to cleaning detergent are not identical, they are similar in size and structure, making the detergent a plausible source of these.	Post-polishing
	Benzene sulfite and related sulfonate, for example 3-Dodecylphenyl hydrogen sulfite	Pre-polishing compound—because benzene and related sulfonates are ingredients of pre-polishing compound used in manufacturing of the polished devices. The hydrogen sulfite moiety of this extractable represents a reduced form of this pre-polishing manufacturing material ingredient.	Pre-polishing
Non-modified	Mixture of hydrocarbons (C32H66 up to C40H82)	Manufacturing materials used pre-cleaning operation (lubricant, aerosol, oil, grease, or gear oil). Such extractables are commonly present in lubricants or greases used during machining operations.	Pre-cleaning

Thirty-seven extractable substances were identified on electropolished articles (Table 3). All assignable organic substances were associated with manufacturing material used in manufacturing steps at or post- the electropolishing operation. None of the known substances could be associated with any pre-electropolishing step. There were twelve substance associations to post-electropolishing manufacturing materials. Nine substances were associated with the cleaning detergent used after the electropolishing operation and three substances were associated with the final packaging.

Eighty-one extractable substances were identified on polished test articles (Table 5). Extractable substances can be associated with manufacturing materials used during pre–polishing, polishing, and post-polishing manufacturing steps. There were thirteen substance associations to post-polishing manufacturing materials, eighteen substance associations to polishing compounds, and twenty-seven associations to pre-polishing compounds.

**Table 5:** Summary of extractables detected above the AET on devices from polished devices group. If an extractable was associated with both pre- and post- mechanical polishing step, both associations are indicated.

Test Article	Total number of organic extractables above AET	Number of extractables associated with manufacturing materials used			Number of extractables not associated with any manufacturing materials
		at the post-polishing operations	at the polishing operation	at the pre-polishing operations	
10th test article	58	12	15	20	28
11th test article	23	1	3	7	14

Eighteen extractable substances were identified on non-modified test articles (Table 6). The following list provides a breakdown of types of manufacturing materials to which extractables were associated: There were four substance associations to manufacturing materials used in post-cleaning operations (packaging materials or plastic sources), three substance associations to manufacturing materials used in cleaning operation (cleaning detergents), and three substance associations to manufacturing materials used pre-cleaning operation (i.e., lubricant, aerosol, oil, grease, or gear oil).

**Table 6:** Summary of extractables detected above the AET on devices from non-modified devices group. If an extractable was associated with both pre- and post-cleaning step, both associations were shown in the table below.

Test Article	Total number of organic extractables above AET	Number of extractables associated with manufacturing materials used			Number of extractables not associated with any manufacturing materials
		at the post-cleaning operations	at the cleaning operation	at the pre-cleaning operations	
12th test article	7	2	0	2	3
13th test article	7	2	0	1	4
14th test article	4	0	3	0	1
15th test article	0	0	0	0	0

## 4 Discussion

The manufacturing of medical devices is a multi-step process that can involve numerous manufacturing materials, including, for example, oils, lubricants, polishing pastes, and cleaning detergents. Complex manufacturing processes utilizing several manufacturing materials can pose a significant challenge for biological safety assessments, as any residues from these materials that may remain on the finished device require thorough evaluation and can lead to extensive testing if existing data are not available to mitigate the risks. By incorporating an electropolishing step into the manufacturing process, the manufacturing material residues that could otherwise introduce local or systemic toxicological risks will be effectively eliminated, thus mitigating overall biocompatibility risks. This can result in an overall risk assessment that waives testing, which is in line with the ethical and regulatory requirements.

The objective of this study was to determine if extractables identified on medical devices were associated with pre-surface modification, surface modification, or post-surface modification steps. Among the five surface modification groups (electropolished, polished, blasted, coated and non-modified devices), two of them (coated and blasted devices) demonstrated a high overall median and range of total exposure. The high median total exposure for coated and blasted group devices relates to their surface complexity. Coated devices include porous coating, which may trap residues of cleaning detergent or different manufacturing materials used after coating process. The surface of blasted devices is typically rougher than polished or electropolished surfaces, and similarly to coated devices, may accumulate manufacturing materials used after blasting operations. The high median total exposure for non-modified devices relates to lack of any step beside cleaning alone which may remove residues of manufacturing materials used during machining operations. The lowest median exposures and low overall exposure ranges were observed for electropolished and polished surface device groups, and warranted further evaluation for implications on device biocompatibility. The objective of this retrospective analysis was to assess whether electropolishing or polishing can effectively mitigate pre-electropolishing or pre-polishing manufacturing material residues.

Electropolishing was identified as one such manufacturing step as it effectively removes 5–10 micrometers (3) from the metallic device surface and, as a consequence, removes all manufacturing residues from the previous steps. Since the electropolishing operation is often employed to create the finished device surface and impart corrosion resistance, it is typically performed at or near the end of the overall manufacturing process, reducing the biocompatibility hazards introduced by manufacturing material residues on the final finished devices. The analyzed extractables from electropolished devices showed no association with pre-electropolishing operations and were traced to manufacturing materials used post-electropolishing. These observations are consistent with electropolishing being a surface reset operation that eliminates upstream manufacturing materials residues. Therefore, this obviates the need for additional testing, including animal testing, to mitigate the hazards associated with the manufacturing materials from the pre-electropolishing step.

The initial analysis indicates that both electropolishing and mechanically polishing devices are capable of modifying the surface and minimizing manufacturing material residues. This is based on low median exposure and a narrow exposure range compared to all other analyzed surface modification groups (Figure 3). Both processes create a smooth surface but differ in the mechanism: Polishing is a mechanical process that uses abrasives to smooth the surface, whereas electropolishing is an electrochemical process that uses an electric current to dissolve surface material completely and thus removes the outermost layer of the device surface. Mechanical polishing smooths the surface through abrasive action, which may reduce surface irregularities and remove some contamination. However, it does not necessarily remove completely the outermost material layer in a uniform manner. As a result, residues from upstream manufacturing steps, particularly hydrophobic contaminants such as oils, may persist because they are only partially removed, redistributed across the surface, or retained within residual surface features. In contrast, electropolishing is an electrochemical dissolution process that removes the outermost surface layer, thereby eliminating residues associated with pre-electropolishing manufacturing operations along with the surface material to which they are adhered. Consistent with this mechanism, extractable residues detected on electropolished devices were associated only with manufacturing steps occurring after electropolishing, whereas mechanically polished devices still showed extractables linked to pre-polishing operations. These findings support the interpretation that mechanical polishing and standard cleaning may reduce residue burden but do not provide the same surface “reset” effect observed with electropolishing.

It is important to note that the analysis of non-modified devices shows that conventional cleaning procedures alone are insufficient to completely eliminate upstream manufacturing residues; in particular, hydrophobic contaminants such as oils and lubricants were not completely removed. These substances adhere strongly to metallic surfaces and can persist even after standard detergent-based cleaning steps. Therefore, additional surface modification techniques, such as electropolishing, are beneficial for removal of these residues and achieving corrosion resistance to support end-use.

While the retrospective analysis provides valuable insights into the effectiveness of electropolishing in “resetting” the device surface through removal of residues from pre-electropolishing operations, several limitations should be acknowledged. First, the final detailed analysis was based on a small and unbalanced sample set, comprising 9 electropolished devices, 2 mechanically polished devices, and 4 non-modified devices out of the 112 datasets initially reviewed. This limits the statistical power of the analysis and the generalizability of the findings. Accordingly, the results should be interpreted as a potential “reset” effect under the conditions studied, rather than as a definitive industry-wide principle. Further studies with larger and more balanced sample sets are needed to confirm broader applicability.

Second, the retrospective nature of the study limits the ability to establish causality with the same rigor as a controlled prospective investigation. Because the analysis relied on historical data, it is difficult to isolate the effect of surface modification from other potentially influential factors, including the effectiveness of cleaning processes, differences in device geometry, and variability among testing laboratories. In this context, the findings should be interpreted as associative rather than causal. In addition, extractables whose chemical identity could not be established with sufficient confidence were excluded from the correlation analysis. While this approach was intended to preserve analytical comparability across datasets, it also represents a limitation, as unknown compounds may contribute meaningfully to the overall extractables profile. If such unknowns were disproportionately represented in one device category, this could influence interpretation of the observed “reset” effect. Among identified extractables, associations with manufacturing materials were made based on chemical relationships, such as shared identities, moieties, or functional groups, and many compounds could not be unequivocally assigned to specific manufacturing sources.

A further limitation of this study is that the electropolished cohort was predominantly composed of stainless steel components, with titanium and cobalt-chromium alloys being comparatively underrepresented. Although electropolishing is based on broadly similar electrochemical principles across metallic biomaterials, the process response and implementation can differ materially by alloy system. Accordingly, the present findings should not be interpreted as establishing equivalent evidence across stainless steel, titanium, and cobalt-chromium devices. In addition, the analysis was restricted electropolished devices that met the following conditions: (i) the raw materials were stainless steel alloys, (ii) electropolishing was performed in accordance with ASTM B912 (3), and (iii) the entire device surface area was exposed to the electropolishing operation. Devices with masked areas or with internal lumens, porous structures, or other complex geometries not amenable to uniform electropolishing were excluded. The observed “reset” effect should therefore be interpreted as applicable to fully exposed electropolished surfaces within the materials and configurations represented in this study. Extension of this conclusion to partially shielded surfaces or more complex device architectures would require further dedicated investigations.

Despite these limitations, the findings suggest that electropolishing may provide substantial value as a surface-resetting step in the biological safety evaluation of medical devices. However, its applicability should be considered within the constraints of the materials, manufacturing conditions, and surface accessibility represented in the present analysis.

## 5 Conclusion

This retrospective analysis of chemical characterization data suggests that under the conditions studied the electropolishing may effectively remove extractable residues associated with upstream manufacturing operations. In this sense, electropolishing may function as a surface “reset” by removing the outermost material layer and thereby reducing the contribution of pre-electropolishing manufacturing residues to the overall extractables profile. Accordingly, for devices with fully exposed electropolished stainless steel surfaces processed under conditions consistent with those evaluated here, biological safety assessments can place greater emphasis on materials and processes used during and after electropolishing. However, these findings should be interpreted within the limitations of the study design, including the small and unbalanced sample set, the retrospective nature of the analysis, and the restricted representation of materials and device geometries. Therefore, the concept of a surface “reset” should be understood here in a scientific and context-specific sense, and broader application would require further investigations. Under defined conditions, this approach could reduce the need for follow-up testing, including animal testing, which is subject to case by case justification.

## Data Availability

The original contributions presented in the study are included in the article/Supplementary Material, further inquiries can be directed to the corresponding author.
