# Supplementary material for: Effectiveness of electropolishing step on elimination of upstream manufacturing material residues on metallic orthopaedic medical device
Source: Front Med Technol. 2026 May 28;8:1803156. doi: 10.3389/fmedt.2026.1803156 (PMC13253740; doi:10.3389/fmedt.2026.1803156)
Supplement: Supplementary file 1 [file Table1.docx]

Supplementary Material

# Supplementary Data: Summary of analysis for extractables detected in electropolished test articles.

The first test article was made from 1.4441 stainless steel. It was manufactured using following process flow: 1.Turning, 2.Brushing, 3.Cleaning, 4.Electropolishing, 5.Paper Grinding, 6.Cleaning, 7.Final inspection, 8.Lasermarking, 9.Passivation, 10.Visual inspection and packaging. Whereas the second test article was made from 1.4441 stainless steel, and it was manufactured using following process flow: 1.Milling, 2.Inspection, 3.Broaching, 4.Milling, 5.Inspection, 6.Rough grinding, 7.Miling, 8.Cleaning, 9.Electropolishing, 10.Paper Grinding, 11.Cleaning, 12.Final inspection, 13.Lasermarking, 14.Passivation, 15.Visual inspection. Both test articles (the first test article and the second test article) are manufactured by the same vendor A. Organic extractables detected above AET and their possible sources for the first and the second test articles are shown in Supplementary Table S1.1.

**Supplementary Table S1.1:** Organic extractables detected above AET and their possible sources for the first and the second test articles. Extractable exposures are in µg/device unit.

| **Extractable** | **Analytical technique** | **Extractable exposure, µg/device** | | **Potential Source of Extractables** | **Association to manufacturing step** |
| --- | --- | --- | --- | --- | --- |
|  |  | **The first test article** | **The second test article** |  |  |
| **Polyethylene glycol containing compound** | LCMS | N/A | 2.6 | The polyethylene glycol containing substance may come from ingredients of alkaline detergent used in final cleaning step. The utilized detergent is a mixture of different lengths of PEG chains and alkyl chains. One ingredient of cleaning detergent associated with polyethylene glycol containing compound is an alkyl-PEG-etherphosphoric acid esters, Na-salt (CAS: 111798-26-6). | Post-electropolishing step -1 for the second test article |
| **Methyl 2-(2-methoxyethoxy)acetate** | LCMS | N/A | 1.2 | The methyl 2-(2-methoxyethoxy)acetate substance likely originates from the alkaline detergent used in final cleaning step. As described above polyethylene glycol compound can originate from one of ingredient (CAS: 111798-26-6) of that cleaner. Therefore, the possible sources of extracted substance are (a) hydrolyzed fragments of PEG which was generated during chemical characterization extraction since this substance is 13% soluble in water [14] or (b) resulting compounds which are produced chemical synthesis reaction of alkyl-PEG-etherphosphoric acid esters, Na-salts. | Post- electropolishing step -2 for the second test article |
| **Methanimidamide, N,N-dimethyl-N'-(4-methylphenyl)** | LCMS | N/A | 0.4 | The methanimidamide, N,N-dimethyl-N'-(4-methylphenyl) substance cannot be associated directly to any contact materials used for manufacturing processes of device. | Not associated - 1 for the second test article |
| **Pentanoic acid, 5,5-dimethoxy-, methyl ester** | LCMS | N/A | 0.2 | The pentanoic acid, 5,5-dimethoxy-, methyl ester substance cannot be associated directly to any contact materials used for manufacturing process of device. | Not associated - 2 for the second test article |
| **Ethanol, 2-(2-ethoxyethoxy)** | GCMS | 1.0 | 0.7 | The ethanol, 2-(2-ethoxyethoxy) substance may come from ingredients of alkaline detergent used in final cleaning step. As described above polyethylene glycol compound can originate form one of ingredient (CAS: 111798-26-6) of that cleaner, which is an alkyl-PEG-etherphosphoric acid esters, Na-salt. Therefore, the possible sources of extracted substance are (a) hydrolyzed fragment of PEG which was generated during chemical characterization extraction or (b) resulting compounds which are produced during chemical synthesis reaction of alkyl-PEG-etherphosphoric acid esters, Na-salts. | Post-electropolishing step -3 for the second test article.  Post-electropolishing step -1 for the first test article |

The third test article was made from 1.4441 stainless steel. It was manufactured using following process flow: 1.Provide raw material, 2.Cut to length, 3.Intermediate cleaning, 4.Make chamber for thread cutting, 5.Thread 15mm unilateral, 6. Intermediate cleaning , 7.Trocar point uniliteral, 8.Deburring edges, 9.Round wire at the end, 10.Electropolish, 11.Final cleaning, 12.Final inspection, 13.Stock parts. The fourth test article was made from 1.4441 stainless steel. It was manufactured using following process flow: 1.Provide raw material, 2.Cut to length, 3.Intermediate cleaning, 4.Trocar point uniliteral, 5.Deburring edges, 6.Round wire at the edges, 7.Electropolish, 8.Final cleaning, 9.Final inspection, 10.Stock parts. The fifth test article was made from 1.4441 stainless steel. It was manufactured using following process flow: 1.Provide raw material, 2.Cut to length, 3.Round wire at the end, 4.Manufacturing of eyelet, 5.Electropolishing, 6.Final cleaning, 7.Final inspection, 8.Stock parts. The third test article, the fourth test article and the fifth test article are manufactured by the same vendor B. Organic extractables detected above AET and their possible sources for the third, the fourth and the fifth test articles are shown in Supplementary Table S1.2.

**Supplementary Table S1.2:** Organic extractables detected above AET and their possible sources for the third, the fourth and the fifth test articles. Extractable exposures are in µg/device unit.

| **Extractable** | **Analytical technique** | **Extractable exposure, µg/device** | | | **Potential Source of Extractables** | **Association to manufacturing step** |
| --- | --- | --- | --- | --- | --- | --- |
|  |  | **The third test article** | **The fourth test article** | **The fifth test article** |  |  |
| **Polyethylene glycol containing compound** | LCMS | N/A | 0.55 | 0.8 | The polyethylene glycol containing substance likely originated from mildly alkaline cleaning detergent used for cleaning after electropolishing operation. Three of the ingredients (CAS: 68425-44-5, CAS: 61791-14-8, and CAS: 26027-37-2) of mild alkaline cleaning detergent are fatty amine ethoxylates. The PEG group, -(EO)_n_H, is present on the one end of ethoxylated fatty acid amide. | Post-electropolishing step -1 for the fourth test article.  Post-electropolishing step -1 for the fifth test article |
| **Hexaethylene glycol** | LCMS | N/A | N/A | 0.4 | Hexaethylene glycol substance and heptaethylene glycol substance may originate from (Poly)ethylene glycol containing compounds discussed above.  Both heptaethylene glycol and hexaethylene glycol may be derived from ethoxylated fatty acid amide. O(EO)_n_H chains may be hydrolized during the extraction in chemical characterization test.  Heptaethylene glycol and hexaethylene glycol may be base ingredients used in synthesis reaction of chemical. The ethoxylated fragments are generated during the synthesis reaction of the fatty amine oxylates, which are ingredients (CAS: 68425-44-5, CAS: 61791-14-8, and CAS: 26027-37-2) of mildly alkaline cleaning detergent used for cleaning after electropolishing. | Post-electropolishing step -2 for the fifth test article |
| **Heptaethylene glycol** | LCMS | N/A | N/A | 0.15 |  | Post-electropolishing step -3 for the fifth test article |
| **Benzoic acid, 2,6-dihydroxy-, octyl ester** | LCMS | 1.0 | N/A | N/A | These substances cannot be associated directly to any contact materials used for manufacturing process of device. Benzoic acid, 2,6-dihydroxy-, octyl ester substance is commonly used in sunscreen. Therefore, the extracted substance may come from laboratory contamination (e.g., lab personnel). | Not associated - 1 for the third test article |
| **Glutaric acid, (cyclohex-3-enyl)methyl 3-methylbut-2-en-1-yl ester** | LCMS | 0.53 | N/A | N/A |  | Not associated - 2 for the third test article |
| **Caprolactam** | LCMS | 0.19 | N/A | N/A | The caprolactam substance is a pre cursor to nylon. It may come from polyamide (PA) bag used as final packaging material for extracted device. | Post-electropolishing step -1 for the third test article. |

The sixth test article was made from 316 LVM stainless steel. It was manufactured using following process flow: 1.Cut to length, 2.Break edge, 3.Trocar point, 4.Degrease, 5.Electropolish, 6.Inspect, 7.Laser etch, 8.Passivation, 9.Package, 10.In-process and final clean, 11.Nitric upgrade, 12.Sterile package and gamma sterile. Organic extractables detected above AET and their possible sources for the sixth test article are shown in Supplementary Table S1.3.

**Supplementary Table S1.3:** Organic extractables detected above AET and their possible sources for the sixth test article. Extractable exposures are in µg/device unit.

| **Extractable** | **Analytical technique** | **Extractable exposure, µg/device, the sixth test article** | **Potential Source of Extractables** | **Association to manufacturing step** |
| --- | --- | --- | --- | --- |
| **7-Aza-B-homostigmastan-6-one, 2,3:22,23-bis[(1-methylethylidene)bis(oxy)]-, (2.alpha.,3.alpha.,5.alpha.,22S,23S)-C35H59NO5** | LCMS | 34.24 | The 7-Aza-B-homostigmastan-6-one, 2,3:22,23-bis[(1-methylethylidene)bis(oxy)]-, (2.alpha.,3.alpha.,5.alpha.,22S,23S)-C35H59NO5 substance is an aminized steroid (Supplementary Figure S.1). This substance cannot be associated directly to any contact materials used for manufacturing process of device.  Since it is not expected to find steroid substances on Zimmer Biomet devices, the chemical characterization laboratory was asked what is the next matching substance for C35H59NO5. The next matching substance had a formula C33H55N3O5 (Supplementary Figure S.2). The exact masses m/z of C35H59NO5 and C33H55N3O5 are 573.439323 and 573.414173. This results in an atomic mass deviation of 0.001377 and 0.026527. The chemical characterization laboratory reports only sum formulas if the deviation from the actual mass is not higher than 50 ppm. This means that the second formula still fits in but has a higher deviation than the first formula. The alternate substance cannot be associated directly to any contact materials used for manufacturing process of device. Considering that this compound is tentatively identified and not expected on the device based on the raw material and contact materials review, it may be misidentified or could be an adduct of the original compound. | Not associated - 1 |
| **3-(3,5-ditert-butyl-4-hydroxyphenyl)propanoic acid octadecyl ester - C35H62O3** | LCMS | 22.10 | The 3-(3,5-ditert-butyl-4-hydroxyphenyl)propanoic acid octadecyl ester - C35H62O3 substance class is often used as a phenol antioxidant in polyolefin plastics. Therefore, the extracted substance may originate from the packaging material (polyurethane pouch). | Post-electropolishing step -1 |
| **Phosphorous acid tris(2,4-ditert-butylphenyl) ester - C42H63O3P** | LCMS | 3.76 | The phosphorous acid tris(2,4-ditert-butylphenyl) ester - C42H63O3P substance is an antioxidant. Antioxidants are a common additive in polyurethane packaging to prevent oxidation. Therefore, the extracted substance possibly originates from the devices’ packaging material (polyurethane pouch). | Post-electropolishing step -2 |


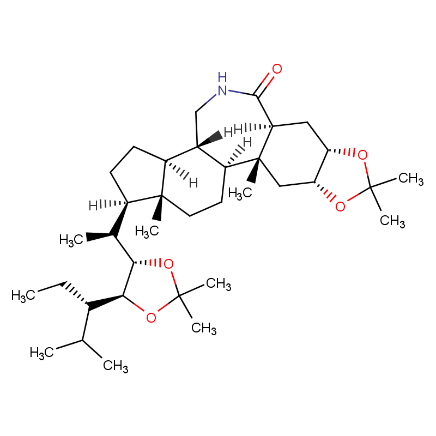


**Supplementary Figure S.1:** Steroid type substance with formula C35H59NO5.


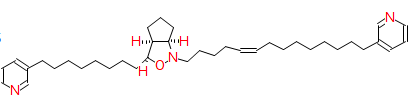


**Supplementary Figure S.2:** Alternative substance with formula C33H55N3O5.

The seventh test article was made from wrought 316L stainless steel. It was manufactured using following process flow: 1.Machine complete, 2.In-process clean, 3.Vibritory finish, 4.In-process clean, 5.Electropolish, 6.In-process clean, 7.Passivate, 8.Final clean, 9.Biological upgrade – nitric acid, 10.Package and steam sterilization. Organic extractables detected above AET and their possible sources for the seventh test article are shown in Supplementary Table S1.4.

**Supplementary Table S1.4:** Organic extractables detected above AET and their possible sources for the seventh test article. Extractables exposures are in µg/device unit.

| **Extractable** | **Analytical technique** | **Extractable exposure, µg/device, the seventh test article** | **Potential Source of Extractables** | **Association to manufacturing step** |
| --- | --- | --- | --- | --- |
| **2-Ethoxyethyl 2-methylbutanoate** | GCMS | 0.75 | The 2-ethoxyethyl 2-methylbutanoate substance is an ethoxylated short chain organic acid. Ethoxylated compounds, especially glycols, alcohols, and fatty acids are used extensively in detergents. The extracted substance may originate from cleaning detergent used after electropolishing. Utilized cleaning detergent is proprietary blend of surfactants and other performance-enhancing ingredients and discloses the possible presence of trace levels of ethylene oxide, propylene oxide and 1,4-dioxane. Therefore, that cleaning detergent contains ethoxylated and alkoxylated species. | Post-electropolishing step -1 |
| **2-Furanmethanol, tetrahydro-5-methyl-, trans-** | GCMS | 0.55 | The 2-Furanmethanol, tetrahydro-5-methyl-, trans- substance cannot be associated directly to any contact materials used for manufacturing process of device. | Not associated - 1 |
| **Oxime-, methoxy-phenyl-** | GCMS | 0.48 | The oxime-, methoxy-phenyl- substance cannot be associated directly to any contact materials used for manufacturing process of device. | Not associated - 2 |

The eighth test article was made from Biodur 108C stainless steel. It was manufactured using following process flow: 1.Machine, 2.Deburr, 3.In-process clean, 4.Electropolish, 5.Dye penetrant inspection, 6.Ultrasonic clean, 7.Final clean, 8. Biological upgrade – nitric acid, 9.Package and steam sterilization. Organic extractables detected above AET and their possible sources for the eighth test article are shown in Supplementary Table S1.5.

**Supplementary Table S1.5:** Organic extractables detected above AET and their possible sources for the eighth test article. Extractable exposures are in µg/device unit.

| **Extractable** | **Analytical technique** | **Extractable exposure, µg/device, the eighth test article** | **Potential Source of Extractables** | **Association to manufacturing step** |
| --- | --- | --- | --- | --- |
| **Unknown RT 0.46** | LCMS | 22.00 | These substances cannot be associated directly to any contact materials used for manufacturing process of device.  Only the hexane extractable unknown at 22.00 µg was present at a level greater than 1.5 µg, which was the dose based threshold (DBT) used for analytical evaluation threshold (AET) establishment. Importantly, this extractable eluted from the analytical column at 0.46 minutes. The analytical column used by external laboratory is a C8 reverse phase column with a column volume of 0.2 ml^[[1]](#footnote-1)^. At a flow rate of 0.3 ml/minute, it would take 0.67 minutes for non-column binding chemical species entering the column to exit the column. In addition, considering binding to a reverse phase column is due to hydrophobic interactions, any compounds passing through the column without binding would be very hydrophilic, and, therefore, water soluble compounds. Considering this unknown species eluted from the column as part of the void volume, it is likely that the chemical is either a column contaminant or a highly water-soluble compound originating from an aqueous based, late manufacturing stage contact material. | Not associated - 1 |
| **Unknown RT 12.47** | LCMS | 0.19 |  | Not associated - 2 |
| **Unknown RT 1.05** | LCMS | 0.19 |  | Not associated - 3 |
| **Tris(2-ethylhexyl) trimellitate <C33H54O6+H>** | LCMS | 0.55 | The tris(2-ethylhexyl) trimellitate substance is a placticizer. However, this substance cannot be associated directly to any contact materials used for manufacturing process of device. | Not associated - 4 |
| **1-Hexanol, 2-ethyl-** | GCMS | 0.45 | The 1-Hexanol, 2-ethyl- may be a fragment of tris(2-ethylhexyl) trimellitate substance which is a placticizer. However, this substance cannot be associated directly to any contact materials used for manufacturing process of device. | Not associated - 5 |
| **Oxygen containing aliphatic compound** | GCMS | 0.95 | These substances cannot be associated directly to any contact materials used for manufacturing process of device. | Not associated - 6 |
| **Methyl α,β-isopropylidene-D-glycerate (structurally similar to)** | GCMS | 0.85 |  | Not associated - 7 |
| **3-Buten-2-ol, 2,3-dimethyl- (structurally similar to)** | GCMS | 0.60 |  | Not associated - 8 |
| **Octadecanoic acid, 2-methyl-, methyl ester, (-)-** | GCMS | 0.55 |  | Not associated - 9 |
| **3-Hexene-2,5-diol** | GCMS | 0.50 |  | Not associated - 10 |
| **3-Hexene, 2-methyl-, (Z)-** | GCMS | 0.48 |  | Not associated - 11 |

The ninth test article was made from forged 22Cr-13Ni-5Mn stainless steel. It was manufactured using following process flow: 1.Turn complete, 2.Degrease, 3.Polish deburr, 4.Electropolishing, 5.Passivation, 6.Ultrasonic clean, 7.Penetrant inspect, 8.Ultrasonic clean, 9.Sonic clean, 10.Penetrant inspect, 11.In-process clean, 12. Laser etch, 13.Final Clean, 14.Biological upgrade – nitric acid, 15. Package and gamma sterile. Organic extractables detected above AET and their possible sources for the ninth test article are shown in Supplementary Table S1.6.

**Supplementary Table S1.6:** Organic extractables detected above AET and their possible sources for the ninth test article. Extractables exposures are in µg/device unit.

| **Extractable** | **Analytical technique** | **Extractable exposure, µg/device, the ninth test article** | **Potential Source of Extractables** | **Association to manufacturing step** |
| --- | --- | --- | --- | --- |
| **Unknown RT 0.64** | LCMS | 2.57 | These substances cannot be associated directly to any contact materials used for manufacturing process of device.  Importantly, only the Hexane unknown eluting at 0.64 minutes and the Ethanol unknown eluting at 0.56 minutes were greater than 1.5 µg, DBT used to establish the study AET. As mentioned above, 0.67 minutes represents the entire void volume of the column used for testing. Considering the void volume retention time represents the minimum amount of time it would take a chemical species that does not bind to the UPLC column matrix to exit the column after injection, any compounds coming out before this would likely have been present on the column (e.g., from a previous analytical run) prior to testing. In addition, as noted above, the column is a reverse phase column, which preferentially binds hydrophobic compounds. Therefore, these unknowns are likely contaminants present in the column or, possibly, in the case of the ethanol extract, a highly water soluble. Considering the electropolishing and cleaning steps would have included copious rinsing, any water-soluble compound residues on the device originating from upstream processes would likely have been removed. Therefore, the unknown compound in the ethanol extract likely originated from analytical column contamination/carry-over or a late manufacturing stage (i.e., post-electropolishing) contact material. | Not associated - 1 |
| **Unknown RT 0.56** | LCMS | 1.71 |  | Not associated - 2 |
| **Unknown RT 8.57** | LCMS | 0.74 |  | Not associated - 3 |
| **Unknown RT 7.94** | LCMS | 0.51 |  | Not associated - 4 |
| **Unknown RT 8.13** | LCMS | 0.37 |  | Not associated - 5 |
| **Unknown RT 6.26** | LCMS | 0.19 |  | Not associated - 6 |
| **Methyl Margarate or isomer <C18H36O2 -H>** | LCMS | 0.41 | The methyl margarate or isomer <C18H36O2 -H> substance cannot be associated directly to any contact materials used for manufacturing process of device. | Not associated - 7 |

# Supplementary Data: Summary of analysis for extractables detected in polished test articles.

The tenth test article was made from cast CoCrMo alloy. It was manufactured using following process flow: 1.Mill, Bore, Deburr, 2.Face/turn/bore, 3.Drill screw hole, 4.In-process clean, 5.Grind, 6.Hone, 7.Solvent Clean, 8.Polish, 9.In-process and final clean, 10.Cold nitric upgrade, 11.Sterile package and gamma sterile. Organic extractables detected above AET and their possible sources for the tenth test article are shown in Supplementary Table S2.1.

**Supplementary Table S2.1:** Organic extractables detected above AET and their possible sources for tenth test article. Extractable exposures are in µg/device unit.

| **Extractable** | **Analytical technique** | **Extractable exposure, µg/device, the tenth test article** | **Potential Source of Extractables** | **Association to manufacturing step** |
| --- | --- | --- | --- | --- |
| **Short Chain Ketones, Aldehydes, and acids** | | | | |
| **Pentanal, 2,2-dimethyl- (14250-88-5)** | GC-MS | 69 | These substances are short chain ketones, aldehydes, and acids. The alkaline cleaning detergent, used during final cleaning (post-polishing operation), was investigated in separate chemical characterization studies to have multiple short chain oxygenated aliphatic compounds, including ketones, esters, and carboxylic acid. These were identified with partial and tentative identification, so other isomers would be plausible. The extracted compounds from this table linked to alkaline cleaning detergent are not identical but they are similar in size and structure. Therefore, alkaline cleaning detergent is a plausible source of these extractables. | Post-polishing operation - 1 |
| **3-n-Propyl-2,4-pentanedione (1540-35-8)** | GC-MS | 11 |  | Post-polishing operation - 2 |
| **3-Isopropyl-5-methylhexan-2-one** | GC-MS | 15 |  | Post-polishing operation - 3 |
| **3-n-propyl-5-methylhexane-2-one** | GC-MS | 6.3 |  | Post-polishing operation - 4 |
| **2-Heptanone, 4,6-dimethyl- (19549-80-5)** | GC-MS | 5.7 |  | Post-polishing operation - 5 |
| **(Hydroxyethyl)methacrylate or isomer/unsaturated polyoxygenated alkyl compound** | UPLC-MS | 0.85 |  | Post-polishing operation - 6 |
| **2-Nonanone (821-55-6)** | GC-MS | 5.3 | These substances cannot be associated directly to any contact materials used for manufacturing process of device. | Not associated - 1 |
| **5-Ethyl-4-tridecanone** | GC-MS | 4 |  | Not associated - 2 |
| **5,8-tridecadione** | GC-MS | 3.4 |  | Not associated - 3 |
| **11-Methoxy-11-oxoundecanoic acid or isomer/unsaturated polyoxygenated alkyl compound** | UPLC-MS | 2 |  | Not associated - 4 |
| **Alkyl Sulfite Esters** | | | | |
| **Sulfurous acid, butyl isohexyl ester** | GC-MS | 45 | These substances are alkyl sulfite esters and they cannot be associated directly to any contact materials used for manufacturing process of device. | Not associated - 5 |
| **Sulfurous acid, butyl hexyl ester** | GC-MS | 3.8 |  | Not associated - 6 |
| **Sulfurous acid, dicyclohexyl ester** | GC-MS | 3.2 |  | Not associated - 7 |
| **Benzene Sulfite and Related Sulfonate** | | | | |
| **3-Dodecylphenyl hydrogen sulfite or isomer/unsaturated polyoxygenated alkyl/aryl sulfur containing compound** | UPLC-MS | 1 | These substances are benzene sulfite and related sulfonate. Ingredients of the pre-polishing tapping compound utilized on tested article are alkylaryl sodium sulfonates (CAS 148520-82-5), which are benzene, mono C10-C13 alkyl derivatives. Therefore, the hydrogen sulfite extractable represents a reduced form of this pre-polishing contact material ingredient. | Pre-polishing operation - 1 |
| **2-(2-Octylphenyl)ethyl methanesulfonate or isomer/unsaturated polyoxygenated alkyl/aryl sulfur containing compound** | UPLC-MS | 2.7 |  | Pre-polishing operation - 2 |
| **Sulfoxide** | | | | |
| **1-Ethoxy-2-[2-(hexylsulfinyl)ethoxy]benzene or isomer/unsaturated polyoxygenated alkyl/aryl sulfur containing compound** | UPLC-MS | 1.7 | The 1-Ethoxy-2-[2-(hexylsulfinyl)ethoxy]benzene or isomer/unsaturated polyoxygenated alkyl/aryl sulfur containing compound is a sulfoxide. Ingredients of the polishing compound, utilized on tested article, contains sulfates and sulfides. Sulfoxides can be obtained as oxidation products of sulfides. Therefore, a plausible source of this extractable is from polishing contact material ingredients. | At the polishing operation - 1 |
| **Organophosphorous Compounds** | | | | |
| **Triphenylphosphine oxide or isomer/unsaturated alkyl/aryl phosphorous containing compound** | UPLC-MS | 6.5 | The triphenylphosphine oxide or isomer/unsaturated alkyl/aryl phosphorous containing compound substance cannot be associated directly to any contact materials used for manufacturing process of device. | Not associated - 8 |
| **Silane** | | | | |
| **Diallylmethylsilane** | GC-MS | 43 | The diallylmethylsilane substance is a silane. It may originate from cured resin which are silicone based. See below (epoxides) for cured resins that could be silicone based instead of epoxide | Pre-polishing operation - 3, or  At the polishing operation - 2, or  Post-polishing operation - 7 |
| **Epoxides** | | | | |
| **Ethanone, 1-(3-ethyloxiranyl)-** | GC-MS | 40 | These substances are epoxides which may originate from cured resin ingredients of contact material utilized to manufacture test article.  Cured resin with no details in Safety Data Sheets are used in: one post-polishing contact materials, three polishing contact materials, and nineteen pre-polishing contact materials. | Pre-polishing operation - 4, or  At the polishing operation - 3, or  Post-polishing operation - 8 |
| **Oxirane, dodecyl (3234-28-4)** | GC-MS | 4.7 |  | Pre-polishing operation - 5, or  At the polishing operation - 4, or  Post-polishing operation - 9 |
| **Aliphatic Amides** | | | | |
| **N,N'-Ethylenebisoleamide or isomer/unsaturated alkyl/aryl amine/amide compound <C38H72N2O2> coeluting with polyoxygenated aliphatic compound** | UPLC-MS | 2.7 | The N,N'-ethylenebisoleamide or isomer/unsaturated alkyl/aryl amine/amide compound <C38H72N2O2> coeluting with polyoxygenated aliphatic compound is aliphatic amide. The ethylene bisoleamide is a known slip agent found in molded, cast, and blown plastics. The source of that extractable could be from any point in the manufacturing process where various plastics are found. For example, containers (e.g., drums) for contact materials, plastic backing for abrasives, and product packaging for product transfer and final packaging. | Post-polishing operation - 10 |
| **Alcohols** | | | | |
| **1-heptanol, 2-propyl- (10042-59-8)** | GC-MS | 6.9 | The 1-heptanol, 2-propyl- (10042-59-8) substance is an alcohol. It is used in the production of lubricants and acrylates for adhesives. The pre-polishing contact material, utilized to manufacture test article, includes mixture of colorants, stabilizers, additives, and lubricants. Therefore, the extracted substance may originate from that pre-polishing contact material. | Pre-polishing operation - 6 |
| **11-Dodecen-1-ol, 2,4,6-trimethyl-,(R,R,R)-** | GC-MS | 4.5 | These substances cannot be associated directly to any contact materials used for the manufacturing process of the device. | Not associated - 9 |
| **5-methyl-5-octen-1-ol** | GC-MS | 4.2 |  | Not associated - 10 |
| **Esters and Polyoxygenated Aliphatics** | | | | |
| **3-methyl-2-butenoic acid, cyclobutyl ester** | GC-MS | 110 | The 3-methyl-2-butenoic acid, cyclobutyl ester substance may originate from alkaline cleaning detergent, used during final cleaning (post-polishing operation). That detergent was investigated in separate chemical characterization studies and shown to have multiple short chain oxygenated aliphatic compounds, including ketones, esters, and carboxylic acid. These were identified with partial and tentative identification, so other isomers would be plausible. The extracted compound from this table linked to alkaline cleaning detergent is not identical but it is similar in size and structure. Therefore, final cleaning detergent is a plausible source of that extractable. | Post-polishing operation - 11 |
| **Dihydro citronellyl angelate** | GC-MS | 11 | These substances cannot be associated directly to any contact materials used for the manufacturing process of the device. | Not associated - 11 |
| **Hexanoic acid, 4-hexadecyl ester** | GC-MS | 7.1 |  | Not associated - 12 |
| **Oxalic acid, cyclohexyl isobutyl ester** | GC-MS | 7 |  | Not associated - 13 |
| **6-Hydroxy-9-oxa-bicyclo[3.3.1]nonan-3-one** | GC-MS | 4.1 |  | Not associated - 14 |
| **Acetic acid, 2-propyltetrahydropyran-3-yl ester** | GC-MS | 3.9 |  | Not associated - 15 |
| **1-propanol, 3-methoxy-2-(methoxymethyl)-2-methyl (20637-34-7)** | GC-MS | 3.4 |  | Not associated - 16 |
| **Oxygen containing aliphatic compound** | GC-MS | 4.6 |  | Not associated - 17 |
| **Ethoxylated Alcohols** | | | | |
| **3,6,9,12,15,18,21,24,27,30-Decaoxaoctatetracontan-1-ol or isomer/aliphatic polyoxygenated compound** | UPLC-MS | 1.3 | These substances are ethoxylated alcohols. They are emulsifiers and wetting agents and can also act as lubricants. Therefore, these compounds could be present in multiple contact materials. While not specifically noted in any contact materials, the following fives are plausible sources:  1.One of the pre-polishing step contact material specifically lists, alcohols C12-13, ethoxylated (CAS 66455-14-9). Since CAS 66455-14-9 is a mixture of alcohols where the average alkyl chain length is 12-13, a C17 chain length ethoxylate would be a plausible species, and, since solubility decreases with alkyl chain length, it would be harder to remove with cleaning than the C12-C13 species.  2. Safety Data Sheet of another pre-polishing compound lists “mixture of colorants, stabilizers, additives, and lubricants.” Ethoxylated alcohols would be plausible lubricants, stabilizers and/or additives.  3. Safety Data Sheet of the next pre-polishing contact material lists a trade secret lubricant, of which ethoxylated alcohols would be a plausible candidate  4.The next pre-polishing contact material lists C9-C11 Alcohol Ethoxylates (CAS 7732-18-5). Considering this is a mixture where C9-C11 are the average alkyl chain lengths, other species would also be present (see above).  5.Safety data sheets of the next pre-polishing compound lists Polyoxyalkylenes, a class of which the extractables identified fall into. | Pre-polishing operation - 7 |
| **2-[2-[2-[2-[2-[2-(2-methylperoxyethoxy)ethoxy]ethoxy]ethoxy]ethoxy]ethoxy]ethanol or isomer/saturated aliphatic polyoxygenated compound** | UPLC-MS | 0.5 |  | Pre-polishing operation - 8 |
| **Phenyl-3,6,9,12,15-pentaoxaheptadecane-1,17-diol or isomer/unsaturated polyyoxygenated alkyl/aryl compound** | UPLC-MS | 2.7 |  | Pre-polishing operation - 9 |
| **Saturated and Monounsaturated Hydrocarbons** | | | | |
| **Cyclopropane, 1,1,2,2-tetramethyl (4127-47-3)** | GC-MS | 41 | These substances are saturated and monosaturated hydrocarbons. Below are potential origins for the whole group of saturated and monosaturated hydrocarbons.  1.Heavy Hydrotreated Naphthenic Petroleum Distillates are used in one of polishing contact materials, and two pre-polishing contact materials, utilized to manufacture test article.  2.Petrolatum is used in polishing contact material, whereas petroleum oil is used in pre-polishing contact material.  3.Mixture of colorants, stabilizers, additives, and lubricants are used in one of pre-polishing contact material.  4.Lubricant is used in one of pre-polishing contact material.  5.Synthetic Isoparaffinic Hydrocarbon (mainly C6 through C13 hydrocarbons) is used in one of pre-polishing contact material.  6.Solvent Refined, Hydrotreated Paraffinic Distillate is used in one of pre-polishing contact material.  7.Distallate (petroleum), hydrotreated heavy naphthenic is used in one of pre-polishing contact material.  8.Paraffin (note, paraffins are saturated hydrocarbons with 22-27 carbons) are used in one of pre-polishing contact material.  9.Mixture of severely hydrotreated and hydrocracked base oil TS-028 is used in one of pre-polishing contact material.  10.Severely Naphthenic Hydrotreated Distillates are used in one of pre-polishing contact materials. | Pre-polishing operation - 10, or  At the polishing operation - 5 |
| **Decane, 5,6-dimethyl-** | GC-MS | 11 |  | Pre-polishing operation - 11, or  At the polishing operation - 6 |
| **Nonane, 4-ethyl-5-methyl** | GC-MS | 11 |  | Pre-polishing operation - 12, or  At the polishing operation - 7 |
| **Tridecane, 3-cyclohexyl- (13151-88-7)** | GC-MS | 8.2 |  | Pre-polishing operation - 13, or  At the polishing operation - 8 |
| **Cyclooctane, tetradecyl (149003-36-1)** | GC-MS | 7.6 |  | Pre-polishing operation - 14, or  At the polishing operation - 9 |
| **Dodecane, 2-cyclohexyl (13151-82-1)** | GC-MS | 7.1 |  | Pre-polishing operation - 15, or  At the polishing operation - 10 |
| **Dodecane, 2-cyclohexyl- (or structurally similar) (13151-82-1)** | GC-MS | 5.6 |  | Pre-polishing operation - 16, or  At the polishing operation - 11 |
| **Cyclohexane, decyl** | GC-MS | 5.3 |  | Pre-polishing operation - 17, or  At the polishing operation - 12 |
| **9-Octadecene, (E)- (7206-25-9)** | GC-MS | 3.9 |  | Pre-polishing operation - 18, or  At the polishing operation - 13 |
| **2,6-dimethyldecane (13150-81-7)** | GC-MS | 3.4 |  | Pre-polishing operation - 19, or  At the polishing operation - 14 |
| **Octane, 5-ethyl-2-methyl- (62016-18-6)** | GC-MS | 3.4 |  | Pre-polishing operation - 20, or  At the polishing operation - 15 |
| **Others** | | | | |
| **1-Hexanol, 2-ethyl-** | GC-MS | 3.7 | The 1-hexanol, 2-ethyl- substance is an alcohol. The alkaline cleaning detergent, used during final cleaning (post-polishing operation), was shown to have multiple short chain molecules with alcohol groups, aliphatic compounds, including ketones, esters, and carboxylic acid. These were identified with partial and tentative identification, so other isomers would be plausible. The extracted 1-Hexanol, 2-ethyl- compound from this table linked to that alkaline cleaning detergent are not identical but they are similar in size and structure. Therefore, final cleaning detergent is a plausible source of these extractables. | Post-polishing operation - 12 |
| **Oxygen containing cyclic compound** | GC-MS | 3.6 | These substances cannot be associated directly to any contact materials used for manufacturing process of device. | Not associated - 18 |
| **unsaturated alkyl/aryl compound (possibly containing nitrogen and/or sulfur)** | UPLC-MS | 65 |  | Not associated - 19 |
| **Unknown RT 4.60** | UPLC-MS | 19 |  | Not associated - 20 |
| **Unknown RT 0.81** | UPLC-MS | 14 |  | Not associated - 21 |
| **Unknown RT 4.75** | UPLC-MS | 8.1 |  | Not associated - 22 |
| **unsaturated polyoxygenated alkyl/aryl amine/amide compound <C9H20N2O6>** | UPLC-MS | 4.9 |  | Not associated - 23 |
| **unsaturated alkyl/aryl amine/amide compound <C36H68N2O2+Cl>** | UPLC-MS | 3.4 |  | Not associated - 24 |
| **unsaturated alkyl/aryl compound (possibly containing nitrogen)** | UPLC-MS | 1 |  | Not associated - 25 |
| **unsaturated polyoxygenated alkyl/aryl amine/amide compound <C37H70N2O4>** | UPLC-MS | 0.55 |  | Not associated - 26 |
| **unsaturated polyoxygenated alkyl/aryl amine/amide compound <C38H70N2O2+Cl>** | UPLC-MS | 0.49 |  | Not associated - 27 |
| **unsaturated polyoxygenated alkyl/aryl amine/amide compound <C18H36N4O8>** | UPLC-MS | 0.42 |  | Not associated - 28 |

The eleventh test article was made from Ti-6Al-4V alloy. It was manufactured using following process flow: 1.Cut Blank, 2.Machine Complete, 3.Fine ceramic blast, 4. Polish, 5.Solvent Clean, 6.In-process and final clean, 7.Cold nitric upgrade, 8.Final clean, 9.Nitric upgrade, 10. Sterile package and gamma sterile. Organic extractables detected above AET and their possible sources for the eleventh test article are shown in Supplementary Table S2.2.

**Supplementary Table S2.2:** Organic extractables detected above AET and their possible sources for eleventh test article. Extractable exposures are in µg/device unit.

| **Extractable** | **Analytical technique** | **Extractable exposure, µg/device, the eleventh test article** | **Potential Source of Extractables** | **Association to manufacturing step** |
| --- | --- | --- | --- | --- |
| **Didodecyl phenyl phosphite (15824-34-7)** | LC-HRMS | 3.3 | The didodecyl phenyl phosphite substance is a packaging, rubber or other plastic component. It may possibly orginate from small diameter abrasive wheel contact materials used during polishing step. | At the polishing operation - 1 |
| **2,2-Dimethylcyclohexyl hydrogen methylphosphonate, TBDMS derivative (NIST#: 273448)** | LC-HRMS | 2 | The 2,2-dimethylcyclohexyl hydrogen methylphosphonate, TBDMS derivative substance is used as lubricant additive and steel corrosion inhibitor which are coming from likely polishing or pre-polishing steps. However, this substance cannot be associated directly to any contact materials used for manufacturing process of device. | Not associated - 1 |
| **Prostaglandins** | | | | |
| **Prost-13-en-1-oic acid, 9,11,15-trihydroxy-6-oxo-, methyl ester, (9α,11α,13E,15S)- (63557-55-1)** | LC-HRMS | 0.95 | These substances are an animal derivative. This can possibly originate from: six pre-polishing contact materials, or four polishing contact materials utilized to manufacture test article. | Pre-polishing operation - 1, or  At the polishing operation - 2 |
| **Prostaglandin F2-α methyl ester (33854-16-9)** | LC-HRMS | 0.6 |  | Pre-polishing operation - 2, or  At the polishing operation - 3 |
| **Quinoline Containing Compounds/Derivatives** | | | | |
| **4-[6-[[6-Methoxy-4-methyl-8-quinolinyl]amino]hexyl]-1-piperazinecarboxylic (83546-83-2)** | LC-HRMS | 1.74 | These substance are quinolones. Although quinolones are found in corrosion inhibitors and antioxidants used in polishing or pre-polishing compounds, these substances cannot be associated directly to any contact materials used for manufacturing process of device. | Not associated - 2 |
| **1-Piperazinehexanamide, N-[6-methoxy-4-methyl-8-quinolinyl]- 3,5-dimethy (83547-07-3)** | LC-HRMS | 0.2 |  | Not associated - 3 |
| **1-(3-Spirocyclohexyl-3,4-dihydro- isoquinolin-1-ylmethyl)-3,3- dimethyl-3,4-dihydro-isoquinoline (NIST#: 301449)** | LC-HRMS | 0.2 |  | Not associated - 4 |
| **Fatty Acids and Fatty Acid Esters** | | | | |
| **Dodecanoic acid 3-Dodecanoic acid 3- (26719-54-0)** | LC-HRMS | 0.4 | These substances are fatty acid and fatty acid esters. Fatty acid esters are not soluble in water and are used in lubricants and metalworking fluids. They possibly originating from pre-polishing contact material, which contains a) Fatty Acid; and b) Fats and Glyceridic Oils, animal, Me esters, mixed with vegetable oils, polymd, oxidized. | Pre-polishing operation - 3 |
| **Benzoic acid, 3-methoxy-, eicosyl (56954-77-9)** | LC-HRMS | 0.3 |  | Pre-polishing operation - 4 |
| **Sebacic acid, tetrahydrofurfuryl tridecyl ester (NIST#: 355729)** | LC-HRMS | 0.6 |  | Pre-polishing operation - 5 |
| **7-Methyloctanoic acid (693-19-6)** | LC-HRMS | 0.2 |  | Pre-polishing operation - 6 |
| **Silanes, Included Halogenated Silane** | | | | |
| **bis[(2E)-Dodec-2-en-1-yloxy](dimethyl)silane (NIST#: 334074)** | LC-HRMS | 0.5 | These substances are silanes, including a halogenated silane. They cannot be associated to any contact materials used before or after the polishing operation.  However, they are common laboratory materials used during the analysis. Since the exposures are relatively low, they can come from contamination of materials used at the testing laboratory. | Not associated - 5 |
| **Silane, diphenylhexadecyloxy(3-methylbut-2-yloxy)- (NIST#: 367652)** | LC-HRMS | 0.2 |  | Not associated - 6 |
| **Silane, methylvinyl(4-methylcyclohexyloxy)methylvinylsilyloxy)nonyloxy- (NIST#: 421128)** | LC-HRMS | 0.2 |  | Not associated - 7 |
| **2,2'-Methylenebis-(6-tert-butyl)-4-ethylphenol, tert-butyldimethylsilyl ether (NIST#: 467074)** | LC-HRMS | 0.2 |  | Not associated - 8 |
| **Phthalates** | | | | |
| **Phthalic acid, monooctyl ester (5393-19-1)** | LC-HRMS | 0.4 | The phthalic acid, monooctyl ester substance is a packaging or other plastic component. It is a plasticizer which may originate from polyurethane bag used as packaging material. | Post-polishing operation - 1 |
| **Ethoxylated Acids/Alcohols** | | | | |
| **3,6,9,12,15,18-Hexaoxanonadecanoic acid, TBDMS derivative (NIST#: 366919) (16142-03-3)** | LC-HRMS | 0.6 | These substances are ethoxylated acids/alcohols, which are used as dispersants, emulsifiers, wetting agents; could be used in any step (pre, polish, and post). They possibly originated from pre-polishing contact material which lists “polyoxyalkylenes;” While these are not alkylenes, they otherwise fit category. | Pre-polishing operation - 7 |
| **Amines Polycyclic Amines** | | | | |
| **Ethylenediamine, N,N,N',N' tetraethyl-1,2-bis(p methoxyphenyl)-(24932-53-4)** | LC-HRMS | 0.4 | These substances are amines and polycyclic amines. Although amines are added as anti-corrosives and pH-buffering agents in metalworking fluids likely in pre-polishing or polishing steps, they cannot be associated directly to any contact materials used for manufacturing process of device. | Not associated - 9 |
| **Pyrimidine, 5-hexyl-2-(4'-pentyl[1,1'-biphenyl]-4-yl)- (92178-46-6)** | LC-HRMS | 0.1 |  | Not associated - 10 |
| **Benzene, 1,2-bis(mesitylaminomethyl)- (NIST#: 152651)** | LC-HRMS | 0.1 |  | Not associated - 11 |
| **Eseroline, 5-(O-methyl)-1-desmethyl-1-(2-menthyloxycarbonyl)-(NIST#: 124264)** | LC-HRMS | 0.7 |  | Not associated - 12 |
| **Others** | | | | |
| **1-(3-Spirocyclohexyl-3,4-dihydro- isoquinolin-1-ylmethyl)-3,3-dimethyl-3,4-dihydro-isoquinoline- C26H30N2** | LC-HRMS | 0.2 | The 1-(3-spirocyclohexyl-3,4-dihydro- isoquinolin-1-ylmethyl)-3,3-dimethyl-3,4-dihydro-isoquinoline substance cannot be associated directly to any contact materials used for manufacturing process of device. | Not associated - 13 |
| **3,6,9,12,15,18- Hexaoxanonadecanoic acid, TBDMS derivative - C19H40O8Si** | LC-HRMS | 0.2 | The 3,6,9,12,15,18- Hexaoxanonadecanoic acid, TBDMS derivative substance cannot be associated directly to any contact materials used for manufacturing process of device. | Not associated - 14 |

# Supplementary Data: Summary of analysis for extractables detected in non-modified test articles.

The twelfth test article was made from Ti-6Al-7Nb alloy. It was manufactured using following process flow:1.Cubic-Rotative Machining (Turning), 2.Cubic Machining, 3.Degreasing, 4.Cleaning, 5.Deburring, 6.Cleaning, 7.Cleaning Hamo, 8.Final Cleaning. Organic extractables detected above AET and their possible sources for the twelfth test article are shown in Supplementary Table S3.1.

**Supplementary Table S3.1:** Organic extractables detected above AET and their possible sources for the twelfth test article. Extractable exposures are in µg/device unit.

| **Extractable** | **Analytical technique** | **Extractable exposure, µg/device, the twelfth test article** | **Potential Source of Extractables** | **Association to manufacturing step** |
| --- | --- | --- | --- | --- |
| **Hexadecanoic acid, butyl ester** | GC-MS | 2.6 | The hexadecanoic acid, butyl ester substance and octadecanoic acid, methyl ester substance, and octadecanoic acid, butyl ester substance are fatty acid esters. Both are used as solvents in wax polishes or as lubricants. However, these compounds cannot be associated directly to any contact materials used for the manufacturing process of device. | Not associated - 1 |
| **Octadecanoic acid, methyl ester** | GC-MS | 1.1 |  | Not associated - 2 |
| **Octadecanoic acid, butyl ester** | GC-MS | 9.1 |  | Not associated - 3 |
| **13-Docosenamide, (Z)-** | GC-MS | 4.6 | The 13-Docosenamide, (Z)- substance is a slip agent, which may possibly come from packaging material. | Post-cleaning operation - 1 |
| **Phenol, 2,4-bis(1,1-dimethylethyl)-** | GC-MS | 0.9 | The phenol,2,4-bis(1,1-dimethylethyl)- substance is likely a fragment of an anti-oxidant like phenol, 2,4-bis(1,1-dimethylethyl)-, phosphite (CAS: 31570-04-4). It can come from any plastic source for example container of detergent. | Post-cleaning operation - 2 |
| **1-Hexadecane** | GC-MS | 0.6 | The 1-Hexadecane substances is an alkane. It is a hydrophobic linear and branched compound, which can come from polishing wax or oil. It is likely possible that it was a part of hydrocarbon “hill”.The hydrocarbon “hill” was detected at high retention times (46 minutes to 52 minutes, corresponding to hydrocarbons C32H66 up to C40H82). The most probable source of this extractable is a heavy oil (heavier than diesel) or a wax. The substance can be possibly associated with following manufacturing materials used at two first machining operation in the process flow: such as lubricant, aerosol, oil, grease, or gear oil. Therefore, extracted substances come from machining steps prior to cleaning. | Pre-cleaning operation - 1 |
| **Hydrocarbon “hill”** | GC-MS | 161 |  | Pre-cleaning operation - 2 |

The thirteenth test article was made from Ti-6Al-7Nb alloy. It was manufactured using following process flow: 1.Cubic-Rotative Machining (Turning), 2.Cubic Machining, 3.Degreasing, 4.Cleaning, 5.Deburring, 6.Cleaning, 7.Final Cleaning. Organic extractables detected above AET and their possible sources for the thirteenth test article are shown in Supplementary Table S3.2.

**Supplementary Table S3.2:** Organic extractables detected above AET and their possible sources for the thirteenth test article. Extractable exposures are in µg/device unit.

| **Extractable** | **Analytical technique** | **Extractable exposure, µg/device, the thirteenth test article** | **Potential Source of Extractables** | **Association to manufacturing step** |
| --- | --- | --- | --- | --- |
| **Hexanedioic acid, bis(2-ethylhexyl) ester** | GC-MS | 0.8 | The hexanedioic acid, bis(2-ethylhexyl) ester substance, hexadecanoic acid, butyl ester substance and octadecanoic acid, methyl ester substance, and octadecanoic acid, butyl ester substance are fatty acid esters. Both are used as solvents in wax polishes or as lubricants. However, these compounds cannot be associated directly to any contact materials used for the manufacturing process of device. | Not associated - 1 |
| **Hexadecanoic acid, butyl ester** | GC-MS | 1.2 |  | Not associated - 2 |
| **Octadecanoic acid, methyl ester** | GC-MS | 0.6 |  | Not associated - 3 |
| **Octadecanoic acid, butyl ester** | GC-MS | 6.9 |  | Not associated - 4 |
| **13-Docosenamide, (Z)-** | GC-MS | 0.6 | The 13-Docosenamide, (Z)- substance is a slip agent, which may possibly come from packaging material. | Post-cleaning operation - 1 |
| **Phenol, 2,4-bis(1,1-dimethylethyl)-** | GC-MS | 0.3 | The phenol,2,4-bis(1,1-dimethylethyl)- substance is likely a fragment of an anti-oxidant like phenol, 2,4-bis(1,1-dimethylethyl)-, phosphite (CAS: 31570-04-4). It can come from any plastic source for example container of detergent. | Post-cleaning operation - 2 |
| **Hydrocarbon “hill”** | GC-MS | 78.7 | The hydrocarbon “hill” was detected at high retention times (46 minutes to 52 minutes, corresponding to hydrocarbons C32H66 up to C40H82). The most probable source of this extractable is a heavy oil (heavier than diesel) or a wax. The substance can be possibly associated with following manufacturing materials used at two first machining operation in the process flow: such as lubricant, aerosol, oil, grease, or gear oil. Therefore, extracted substances come from machining steps prior to cleaning. | Pre-cleaning operation - 1 |

The fourteenth test article was made from Ti-6Al-4V alloy. It was manufactured using following process flow: 1.Cubic-Rotative Machining (Turning), 2.Degreasing, 3.Cleaning, 4.Cubic Machining, 5.Degreasing, 6.Cleaning, 7.Cleaning, 8.Cleaning Hamo, 9.Final Cleaning. Organic extractables detected above AET and their possible sources for the fourteenth test article are shown in Supplementary Table S3.3.

**Supplementary Table S3.3:** Organic extractables detected above AET and their possible sources for the fourteenth test article. Extractable exposures are in µg/device unit.

| **Extractable** | **Analytical technique** | **Extractable exposure, µg/device, the fourteenth test article** | **Potential Source of Extractables** | **Association to manufacturing step** |
| --- | --- | --- | --- | --- |
| **1-Decanol, 2-hexyl-** | GC-MS | 1 | The 1-Decanol, 2-hexyl- substance can be fragment of ingredient of final cleaning detergents. Both cleaning detergents include in their composition decan-1-ol, ethoxylated. Therefore, extracted substance is possibly originating from final cleaning detergents. | At cleaning operation - 1 |
| **Valine, N-[N-[3-[(carboxymethyl)thio]-Ndecanoyl-L-alanyl]glycyl]-, dimethyl ester, L-** | LC-HRMS | 0.5 | This compound cannot be associated directly to any contact materials used for the manufacturing process of device. | Not associated - 1 |
| **Dodecylbenzenesulfonic acid** | LC-HRMS | 3.5 | The dodecylbenzenesulfonic acid substance is derivative of benzenesulfonic acid, 4-C10-30-sec-alkyl derivatives substance (CAS: 85536-14-7) which is ingredient of final cleaning detergent. Therefore, extracted substance likely originates from final cleaning detergents. | At cleaning operation - 2 |
| **Polyethylene glycol compound** | LC-HRMS | 2.2 | The polyethylene glycol compound may come from ingredients of detergents used in final cleaning step. Ingredients of the first cleaning detergent associated with polyethylene glycol compound are: decan-1-ol, ethoxylated (CAS: 26183-52-8); alcohol, C9-11-iso, C10-rich, ethoxylated (CAS: 78330-20-8); and ethoxylated coco fatty amine (CAS: 61791-14-8). Ingredients of the second cleaning detergent associated with polyethylene glycol compound are: decan-1-ol, ethoxylated (CAS: 26183-52-8); ethoxylated coco fatty amine (CAS: 61791-14-8). Therefore, extracted substance likely originates from final cleaning detergents. | At cleaning operation - 3 |

The fifteenth test article was made from Ti-6Al-4V alloy. It was manufactured using following process flow: 1.Material from stock, 2.Turning, 3.Cleaning, 4.Laser Marking, 5.Final Cleaning, 6.Final Control, 7.Packaging and Final release, 8.Shipment, 9.Cleaning Hamo. No organic extractables was detected during chemical characterization study of fifteenth test article.

1. Based on the column manufactures specifications for the Acquity UPLC BEH C_8_, 1.7µm particle size, 50 mm length, 2.1 mm internal diameter column used; See *Acquity UPLC and Acquity Premier BEH Columns, Care and Use Manual.* Waters Corporation, Rev K, 2021. [↑](#footnote-ref-1)
